# Assessing participants’ experiences with the COVID-19 symptom diary in a clinical trial

**DOI:** 10.1186/s41687-025-00901-5

**Published:** 2025-08-11

**Authors:** T. Michelle Brown, Chisom Kanu, Magdalena Harrington

**Affiliations:** 1 RTI Health Solutions, Research Triangle Park, NC USA; 2 Global Access & Value, Pfizer Inc, 66 Hudson Blvd East, Ste 20, New York, NY 10001 USA

**Keywords:** COVID-19, SARS-CoV-2, Patient-reported outcomes, Exit interviews, Clinical trial

## Abstract

During a phase 2/3 study of twice-daily nirmatrelvir 300 mg coadministered with ritonavir 100 mg versus placebo for 5 days (NCT05011513), participants reported presence and severity of COVID-19 symptoms using a COVID-19 symptom diary in accordance with FDA guidance. Here, we aimed to evaluate the content validity of the COVID-19 symptom diary and global impression items through qualitative interviews as recommended by the FDA. Study participants kept a daily electronic diary to record the presence and severity of 14 COVID-19 symptoms (stuffy or runny nose, sore throat, shortness of breath, cough, low energy or tiredness, muscle or body aches, headache, chills/shivering, feeling hot or feverish, nausea, frequency of vomiting, frequency of diarrhea, sense of smell, and sense of taste). They also answered questions regarding 3 global impression items (return to health, return to usual activities, and overall symptom severity). A subset of clinical trial participants was interviewed at study completion to assess content validity of the diary. Overall, 25 participants were interviewed across 10 clinical sites. Each of the 14 symptoms included in the diary were reported by ≥ 1 participant, with low energy/tiredness being reported most frequently (88.0%). Twelve participants (48.0%) reported ≥ 1 symptom that was not captured by the diary, with dizziness being the most common (12.0%). Rates of these symptoms were lower (≤ 12.0%) than the 14 prespecified symptoms included in the diary (16.0−88.0%). Based on participant feedback, all 14 diary items were clear and generally easy to answer using the response scales, and all were interpreted as intended. The 3 global items received similar feedback, although a few participants noted that there were challenges in interpreting the items, describing questions as “vague” and “confusing”, or that responses were too limited; one-third reported having difficulty calculating overall symptom experience for multiple symptoms. Participants reported that the electronic application for the diary and global items was easy to use and enabled the items to be easily completed. Exit interview results support the content validity of the COVID-19 symptom diary and global impression items, with the 14 symptoms included in the diary reflecting the most commonly experienced COVID-19 symptoms. NCT05011513; https://classic.clinicaltrials.gov/ct2/show/NCT05011513.

**Supplementary Information:**

The online version contains supplementary material available at 10.1186/s41687-025-00901-5.

## Background

COVID-19 continues to be a public health threat. In the United States, COVID-19 was responsible for 1.2 per 100,000 population weekly hospital admissions and 0.7% of deaths from April 20‒27, 2024 [1].

The oral antiviral nirmatrelvir administered with ritonavir is a SARS-CoV-2 main protease (M^pro^) inhibitor approved for treatment of mild-to-moderate COVID-19 in adults at high risk for severe disease [2]. Two phase 2/3 Evaluation of Protease Inhibition for COVID-19 (EPIC) trials evaluated nirmatrelvir/ritonavir efficacy [3, 4]. For the primary endpoint of time to sustained alleviation of COVID-19 symptoms in the EPIC trial in people at standard risk for severe disease (EPIC-SR), participants recorded presence and severity of their COVID-19 symptoms in electronic diaries [3]. They also answered global impression questions regarding return to health and activities and overall symptom severity through the electronic application.

To evaluate the content validity and comprehensibility of the COVID-19 symptom diary and global impression items used in the EPIC trials, qualitative interviews were conducted among a subset of EPIC-SR participants, consistent with US Food and Drug Administration (FDA) guidance [5].

## Materials and methods

### Study population and setting

The phase 2/3, double-blind, randomized, placebo-controlled EPIC-SR study (NCT05011513; August 25, 2021, to July 25, 2022) has been described previously [3]. Briefly, eligible participants had confirmed SARS-CoV-2 and were either fully vaccinated against COVID-19 with ≥ 1 risk factor for severe disease or unvaccinated (or not vaccinated within the past 12 months) with no risk factors. Participants were randomized 1:1 to receive twice-daily nirmatrelvir 300 mg and ritonavir 100 mg or placebo for 5 days. As part of the primary study objective, participants used an electronic diary, prespecified by FDA guidelines, to record presence and severity of COVID-19 symptoms daily (Table 1) [3, 5]. Diaries were completed from Days 1‒28 using an electronic application on their personal or trial site device. The electronic application sent daily reminders to complete diary entries within fixed windows (i.e., 24 h) to ensure accurate representation of their experience during that time. The diary comprised 14 symptom-related questions; 3 additional global impression items were also available on the application.

**Table 1:** COVID-19 symptom diary^a^ and global impression items

	Response options
Symptom diary items	
Severity	
1. Stuffy or runny nose	• None (I did not have this symptom) • Mild • Moderate • Severe
2. Sore throat	
3. Shortness of breath (difficulty breathing)	
4. Cough	
5. Low energy or tiredness	
6. Muscle or body aches	
7. Headache	
8. Chills or shivering	
9. Feeling hot or feverish	
10. Nausea (feeling like need to throw up)	
Frequency	
11. How many times did you vomit (throw up) in the last 24 h?	• I did not vomit at all • 1–2 times • 3–4 times • 5 or more times
12. How many times did you have diarrhea (loose or watery stools) in the last 24 h?	• I did not have diarrhea at all • 1–2 times • 3–4 times • 5 or more times
Sensory	
13. Rate your sense of smell in the last 24 h	• My sense of smell is THE SAME AS usual • My sense of smell is LESS THAN usual • I have NO sense of smell
14. Rate your sense of taste in the last 24 h	• My sense of taste is THE SAME AS usual • My sense of taste is LESS THAN usual • I have NO sense of taste
Global impression items	
15. In the past 24 h, have you returned to your usual health (before your COVID-19 illness)?	• Yes • No
16. In the past 24 h, have you returned to your usual activities (before your COVID-19 illness)?	• Yes • No
17. In the past 24 h, what was the severity of your overall COVID-19–related symptoms at their worst?	• None • Mild • Moderate • Severe

^a^Participants were asked “What was the severity of your [insert symptom] at its worst over the past 24 hours?”

Note This version of the symptom diary and global impression items was used in the EPIC-SR trial (Evaluation of Protease Inhibition for COVID-19 in Patients at Standard Risk for Progression to Severe Disease); it includes a minor modification to that described in the source, specifically the addition of “I did not have this symptom” to the “None” response option for items 1–10

Symptoms related to COVID-19 were collected based on the prespecified US Food and Drug Administration guidelines [5]

In this protocol-specified analysis, exit interviews were conducted among a subset of EPIC-SR participants to evaluate participant understanding of diary symptom and global impression items. Participants were selected from study sites with high expected participant volumes. Once site staff were trained, they invited all subsequent trial participants at their sites to participate in interviews. Eligible participants were ≥ 18 years of age; consented to trial participation (overall study consent included qualitative interviews); completed treatment and Day 34 study visit (i.e., end of safety follow-up period); and could read, speak, and understand English.

Up to 50 individual interviews were planned; fewer interviews could be deemed sufficient based on interim results (i.e., clear and consistent patient feedback, with no novel information generated per additional interview after the first 20 − 25 interviews, signifying data completeness).

### Interviews

Interviews were conducted within 10 business days after the Day 34 visit via telephone by experienced qualitative interviewers using a semi-structured guide (Supplemental Appendix). The 45- to 60-minute interviews began with a brief study overview and a few open-ended questions asking participants to describe their initial symptoms and how they were diagnosed with COVID-19. General questions were followed by more targeted questions designed to gather information about specific symptoms included in the diary. Participants were then asked about their understanding and ease of response to the questions and their perceived relevance of each of the COVID-19 symptoms and global impression items. Additional information on interview procedures and adverse event reporting are provided in the Supplemental Appendix.

### Analysis

Thematic analysis methods using field notes and transcripts were used to analyze interview data. Coding and analysis processes were facilitated using Excel. Qualitative analysis output included a summary of codes relating to participants’ COVID-19 experiences, focusing on evaluating diary comprehensiveness. Where appropriate, descriptive summaries were accompanied by frequency counts of numbers of participants reporting a given finding. Quotes from transcripts best representing the themes identified in the text were noted.

Key concepts and leading trends were identified in each interview, and individual results were compared across interviews allowing for identification of themes or patterns in the responses. Formal hypotheses were not tested in this qualitative analysis. Descriptive statistics, as appropriate (e.g., mean, standard deviation, range), including those for frequency (e.g., number, percentages) of select themes from the qualitative data, were computed.

## Results

### Participants and COVID-19 symptoms experiences

**Fig. 1:**
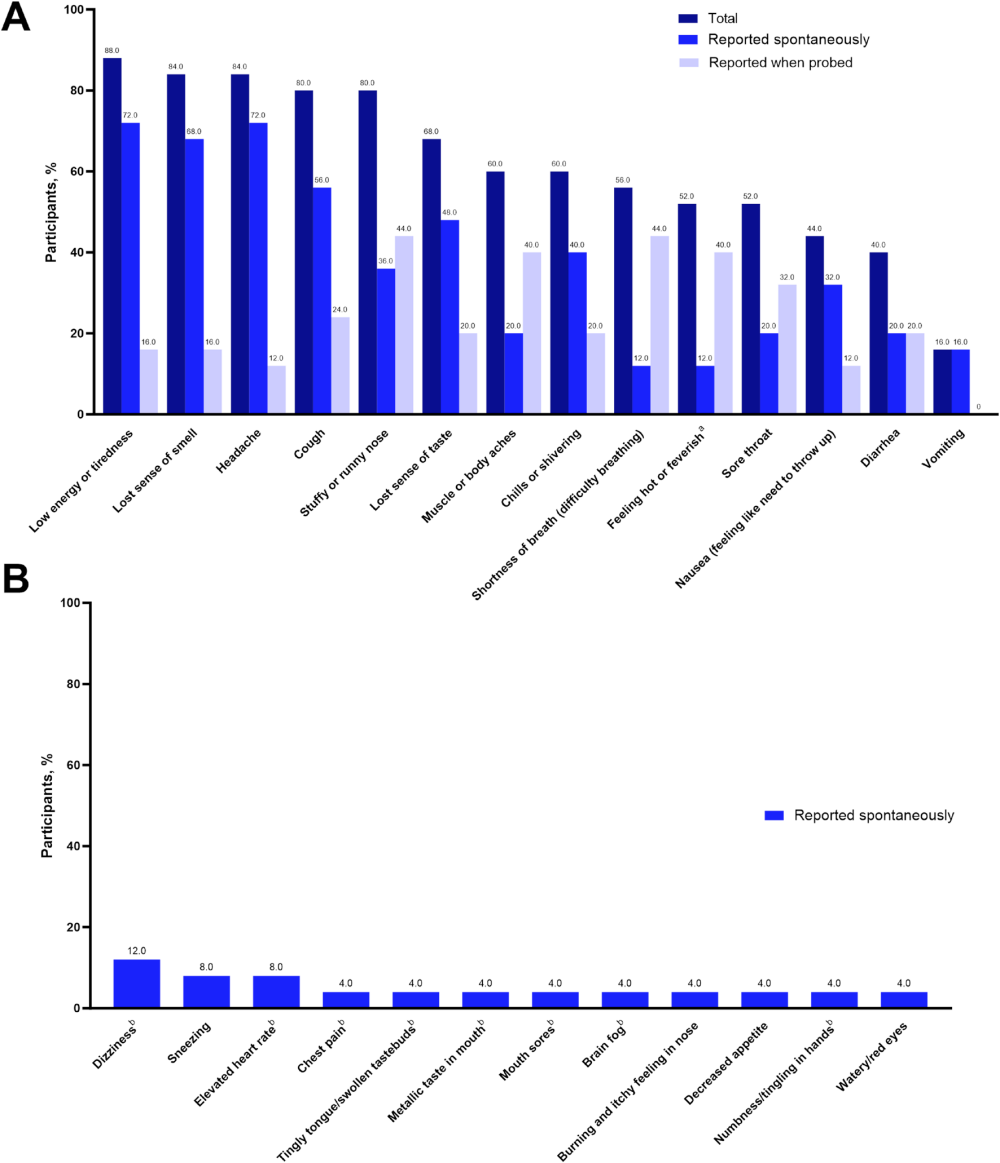
COVID-19 symptoms reported by participants (**A**) from the diary or (**B**) as additional symptoms not captured in the diary. ^a^Ten participants spontaneously reported a fever before endorsing feeling hot or feverish when probed. ^b^Adverse event reports were sent to the clinical sites for this reported experience

Details on site participation and interview conduct are described in the Supplemental Appendix. Overall, 25 EPIC-SR trial participants (mean age: 35.2 years; 64.0% female) were interviewed across 10 sites. The 14 symptoms included in the diary were those most frequently endorsed by interviewees among all reported symptoms. Each was reported by ≥ 1 participant, with full endorsement by the seventh interview, based on spontaneous report, and by the second interview when both spontaneous and probed responses were considered. The most commonly reported symptom was low energy/tiredness (total, 88.0%: reported spontaneously, 72.0%; reported when probed, 16.0%; Fig. 1A). Twelve participants (48.0%) reported ≥ 1 symptom not captured by the diary, most commonly dizziness (12.0%; Fig. 1B).

Twenty participants (80.0%) provided feedback on the most bothersome symptoms (Supplementary Table S1). The most frequently reported included low energy/tiredness in 5 participants, lost sense of taste (*n* = 4) or smell (*n* = 3), headache (*n* = 3), and cough (*n* = 2). Four participants noted that their most bothersome symptom (chest pain, visibly swollen taste buds, dizziness, and burning/itchy feeling in the nose [*n* = 1 each]) was not captured by the diary; 1 participant was unable to identify any symptom as particularly bothersome.

### Cognitive debriefing of the COVID-19 symptom diary

After providing an exhaustive listing of COVID-19 symptoms, participants provided additional information on their experiences and feedback on diary items as part of the debriefing module of the interview. Examples of phrases used by participants to describe symptoms in their own words are summarized in Supplementary Table S2. Based on participant feedback, all 14 symptoms were clear and interpreted as intended (Table 2). When debriefing, participants found each diary item to be clear and generally easy to answer using the response scales provided, indicating they were able to easily respond to each item. For the 10 severity items, 20/25 participants (80.0%) did not note any issues with severity item response options. One participant (4.0%) noted difficulty in rating the severity of a stuffy nose, and 4 participants (16.0%) noted feeling limited by the response scale, suggesting expanded response options to capture more detailed summaries of their symptom experiences (additional details in Table 2). Most (92.0%) participants found identifying their worst experience of each symptom in the past 24 h easy. Two participants had some difficulties identifying their worst experience of stuffy nose and low energy/tiredness because these symptoms were brief and intermittent, but were still able to provide responses.

**Table 2:** Comprehensibility of the symptom diary concepts

Item	Summary of feedback
Symptoms, severity items	
• Stuffy or runny nose • Sore throat • Shortness of breath (difficulty breathing) • Cough • Low energy or tiredness • Muscle or body aches • Headache • Chills or shivering • Feeling hot or feverish • Nausea (feeling like need to throw up)	All concepts were understood as intended, with all participants finding this item easy to understand and answer based on identifying symptoms at their worst over the past 24 h and using the categorical severity response scale provided. For the stuffy or runny nose item, although 1 participant mentioned that it was challenging to decide whether a severe stuffy nose that lasted for 5 min was actually moderate or severe in a 24-hour time span, no changes were suggested by participants to improve this or other items or make them clearer. Participants found this scale easy to use for rating the severity of their symptoms, describing it as “user friendly” and “easy to use.” However, 4 participants (16.0%) noted feeling limited by the response scale, preferring a 5-point verbal response scale (to more easily identify the scale’s midpoint) or a 0- to 10-point numeric rating scale (to capture degrees of symptom severity). The following quotes represent these participants’ feedback on the response scale: *-Yes*,* between moderate and severe I would say it was a little bit harder to decipher which one I should pick. I knew that I felt sometimes it was more severe because I would go to eat something*,* and I would instantly lose my lunch. So*,* I would pick severe for that one kind of thing. And if I didn’t instantly lose it*,* I would pick moderate. So*,* I’m not sure if that was the right way to pick my options.* *-Because even an 8 from a 10 is such a huge difference. They are still severe but to me*,* it would have made a huge difference to be able to say yes*,* it was severe*,* but I got 3 dishes washed. As opposed*,* I just could not roll myself out of bed.* *-I think the rating scale probably should have had 5-ish options. I felt sort of limited and a little bit like I was having difficulty deciding between does it feel more mild or moderate for some items…I would want to have seen a section that was like*,* was this better or worse than yesterday*,* than the prior 24 h. Or was it an improvement from the day before or same or worse. Because there were many days where I would put mild in a row*,* but there were still fluctuations.*
Symptoms, frequency items	
• Vomiting • Diarrhea	Both concepts were understood as intended, with all participants finding this item easy to understand and answer using the 4-point frequency rating scale. The rating scale options ranging from “I did not vomit at all” to vomiting “5 or more times” or from “I did not have diarrhea at all” to having diarrhea “5 or more times,” respectively, were also considered appropriate and distinct from one another. Participants shared that it was easy to recall episodes of diarrhea and keep track of the number of episodes. For the vomiting item, 1 participant mentioned that it could be a little difficult to answer due to uncertainty about whether dry heaving was considered vomiting and because one might not be able to accurately remember the number of vomiting episodes if there were multiple incidents in a 24-hour period. However, these issues did not affect the participant’s ability to answer the item. Besides this participant’s suggestion to expand the definition for vomiting in parentheses to specify that food/fluid was actually expelled, no other changes were proffered by other participants to improve this item or make it clearer. For the diarrhea item, no changes were suggested by participants to improve this item or make it clearer.
Symptoms, sensory items	
• Sense of smell • Sense of taste	This concept was understood as intended, with all participants finding this item easy to understand and answer using a 3-point rating scale ranging from “My sense of smell is THE SAME AS usual” to “I have NO sense of smell” or from “My sense of taste is THE SAME AS usual” to “I have NO sense of taste.” Three participants who lost their sense of smell felt that the response options for this item were inadequate because they did not capture day-to-day variations of alterations in smell (e.g., coffee not smelling like coffee, spices smelling different). One participant suggested including an open-ended response option to allow for entry of additional details. Another participant said a numeric rating scale might better capture variations in this sense for those who did not completely lose this sense. However, these participants were able to provide a response to this item using the “less than usual” option to describe all variations in the sense of smell, which were less than they would normally experience but not completely absent. No other changes were suggested by participants to improve this item or make it clearer. Some participants who experienced loss of their sense of taste felt that the response options for this item were inadequate as they did not capture day-to-day variations in taste/minor improvements or alterations in taste (e.g., coffee tasted terrible, tea tasted like water, juice tasted different). One participant suggested an open-notes field to capture additional information about this symptom. Another participant mentioned that a numeric rating scale might be more appropriate to capture variations in the sense of taste for individuals who did not completely lose this sense. No other changes were suggested by participants to improve this item or make it clearer.
24-hour recall period	Most participants (*n* = 20/25; 80.0%) said that it was easy to recall the severity of the diary symptoms they experienced in the prior 24 h. The remaining 5 (20.0%) participants were usually able to easily select their response based on the past 24 h but commented on why it was sometimes challenging for them to recall the past 24 h. One patient said he was very sick and had difficulties keeping track of time (i.e., was foggy brained), especially when paired with extensive amounts of sleep and isolation. The remaining 4 participants stated it was easier to recall the past 24 h for the severe symptoms or those that lingered compared with those that were mild, intermittent, or short-lived. The following quotes depict these participants’ feedback on the recall period: • *For the things that were more on the mild side*,* I would say it was a little more difficult because it didn’t cross my mind as I was doing it. So*,* sometimes I would have to go and hit the back button because I would think*,* okay*,* actually yeah*,* this morning I woke up coughing. But it was easy for the things that were more severe like the tiredness and like the nausea and things like that.* • *Well*,* I think just in general*,* all of them were difficult to keep track of because during the whole time you have very foggy brain. Time kind of just doesn’t really exist because you are so isolated.* • *Stuffy nose. That one’s hard to judge…because that was kind of off and on…that’s hard to remember if my nose was stuffy or not.* • *Well*,* there may have been a little bit of nausea and since it hadn’t lasted very long*,* you’d forget about it.*
Diary comprehensiveness and overall impressions	All interview participants reported their generally positive overall impression of the diary sharing that it was self-explanatory, easy to complete, comprehensive, and adequately captured their COVID-19 experience. The following quotes depict these participants’ feedback on the overall impression: *-It [the diary] was very self-explanatory. I didn’t feel like I needed clarification*,* or I didn’t need to call someone and say*,* what do you mean? What do you want from this? I don’t understand. I felt very well understood. Open the phone*,* look at each question*,* and knowing what was expected of me.* *-I think the diary did a great job of covering all the symptoms. There are some symptoms I wasn’t even aware were symptoms of COVID-19. So*,* it was very thorough.* *-I think it [the diary] described all the symptoms that I experienced and then some that I never experienced. I don’t think there would have been other things that should have been added*,* I think it covered everything.* Seven participants (28.0%) said the diary could have included some additional symptoms that were not included in the diary, including dizziness, sneezing, red/watery eyes, burning/itchy sensation in nose, congestion, and chest pain. Additionally, 10 participants mentioned that the diary could have captured more detail on the symptoms it measured to provide a better picture of their experience, making suggestions such as updates to the response scales (e.g., use of a numeric rating scale for the symptom severity items, addition of an “Other” response option) and the inclusion of an open-ended section to capture symptoms outside those included in the diary. The following quotations reflect some participants’ wishes for the diary to include more detail and options for custom comments: • *I felt at a few times that I could have used a notes section to explain some of my answers or qualify them a little bit better.* • *I feel like maybe there could have been a little more*,* I guess questions that went further into certain symptoms*,* like the body aches or the fatigue…I feel like it could have asked me whether or not I was fatigued a certain amount…if my fatigue went away yesterday after doing something specific*,* or how long did I feel the fatigue for.* • *I think you would answer all the questions that you presented and then there’d be an extra one that would say “are there any symptoms not described*,* and then what are those.” And then maybe the severity of it.* • *Yes*,* just like a blank area. That’s maybe even an option that you could taste or smell certain items. And then you could have a box for what items*,* are the specific items you could or could not taste or smell if it was less than normal?*

For the 2 frequency items, 1 participant (4.0%) suggested expanding the definition for vomiting in parentheses to specify that food/fluid was actually expelled (to further distinguish vomiting from nausea). For the 2 sensory items, participants who experienced loss of smell or taste felt that the response options did not capture day-to-day variations or improvements in the respective taste.

Although participants stated that the diary adequately captured their COVID-19 experiences, 7 (28.0%) indicated additional symptoms (e.g., dizziness, sneezing, congestion) should be included. Some participants provided suggestions for the diary including use of open-ended items and comment fields.

Most (80.0%) participants found it easy to recall the severity of diary symptoms during the previous 24 h (Table 2). Five (20.0%) were able to easily select their response based on the past 24 h but noted why it was sometimes challenging for them to recall the previous 24 h, with 4 (16.0%) mentioning it was easier to recall severe symptoms compared with those that were mild, intermittent, or short-lived.

Most participants noted that the 3 global impression items were easy to answer and perceived as clear, and the items were interpreted as intended (Table 3). For the *return to usual health* item, challenges in interpreting and selecting a response were noted, with the question described as “vague” and “confusing” and having limited response options. Two participants commented that they could not distinguish between the *return to usual health* and *return to usual activities* items. For the *average symptom severity* item, 8 (32.0%) participants expressed difficulty calculating their overall symptom experience for multiple symptoms at their worst and over the past 24 h.

**Table 3:** Cognitive debriefing of global impression items and electronic device usability

Item or device	Summary of feedback
Return to usual health	Participants generally interpreted this item as the resolution and absence of all COVID-19 symptoms that affected how they felt and their ability to perform daily activities. Most participants were able to easily recall their health in the prior 24 h, compare it to how they felt before developing COVID-19, and easily provide a yes or no response. Two participants (8.0%) elaborated on the uncertainties associated with this question. Specifically, one felt the question was a bit confusing after she had responded with “Yes” the first time since in the following days her return to usual health was over a period longer than 24 h. This participant suggested that the format of this question should have changed once a “Yes” response was provided to possibly exclude the reference to the “past 24 h.” Another participant felt that this question was hard to answer in the presence of residual/lingering symptoms due to the limited response options, suggesting a more robust response scale. The following quotations reflect some participants’ feedback: • *I was confused because it said in the past 24 h. So*,* the first time that I felt like*,* yes*,* I have returned to my usual health*,* I said*,* yes. And then the next day*,* I wasn’t sure if I should say yes or no*,* because when I returned to my usual health wasn’t…it was no longer within the past 24 h.* • *I started feeling better and I started feeling really good*,* mentally and physically*,* but I still had a stuffy nose and I still had a cough*,* but I was back to my normal healthy feeling*,* but I still had some leftover symptoms. So*,* I kind of didn’t know whether to say yes or no because I still had a symptom*,* but I felt fine.*
Return to usual activities	Participants generally interpreted this item as their ability to perform everyday activities (e.g., walking their dogs, doing dishes, making food, going to work/school) For some patients with lingering symptoms, they would be able to return to their usual activities before they could return to their usual health. As such, patients perceived the usual health and usual activities questions as different. All were able to easily recall their activities in the past 24 h and compare it with their pre–COVID-19 ability, and they found it easy to answer this item using the response options provided.
Average symptom severity	Participants generally understood the item asking about the severity of their overall COVID-19–related symptoms as intended and found it easy to recall their symptom experiences over the past 24 h. Although 8 participants (32.0%) said this item was more challenging to answer than other items, all participants said they were able to provide a response using the response options provided. Participants generally found the response scale easy to use and were able to distinguish between the response options ranging from “None” to “Severe.” Two participants mentioned some issues with the response options: one said that it was challenging to differentiate between some of the response options because their interpretation was subjective, and the other participant suggested including additional response options at the lower end of the response scale because he felt limited by the available options. Example quotations for how some patients found this item more challenging to answer are provided below: • *Yeah*,* so for that one I would say it was a little more difficult because again I was still feeling kind of off like with the nausea and the tiredness. But again*,* I wasn’t sure if it was COVID related or if it was just from being home for 10 days and at least with the tiredness.* • *…like let’s say I felt 5 symptoms and then only 2 of them were severe*,* but the other 2 were mild*,* so is that considered moderate? So*,* I tried to answer on the worst*,* whatever the worst was for the day. So*,* if my worst symptom*,* regardless of whether it was 1 or 5*,* it’s…5 symptoms. If it was still 1 of them that was severe*,* I still reported severe.* • *Sort of when considering all the different symptoms that I had experienced that day and trying to balance or average them out*,* and also my*,* just my overall feelings of illness. I tried to rate it based on that kind of my general impression of how did I feel today. That was a little bit harder to answer because when I started thinking about the overall severity*,* it also brought to mind kind of the overall population of people who’ve had COVID and who have been hospitalized and all that kind of stuff.*
Electronic device usability	All participants found it easy to learn how to use the app and easy to complete the diary in the app regardless of the device they used to access the app, describing it as “intuitive” and “as easy as it gets.” Although not asked directly, many generally described completion of the diary as quick and easy (e.g., 2–3 min, 3–4 min), and no participant reported it as burdensome. Five participants made minor suggestions to improve the app’s usability, such as the addition of an audio reminder in the app to better draw attention to completing the diary, having the app transition to the next question once a response was provided without the respondent having to hit the “NEXT” button, and having the app keep a count of which day’s questions were available/being completed to avoid multiple diary completions in one day.

Overall, 17 (68.0%) participants used the electronic app on their personal smartphones and 8 (32.0%) used a second device provided by their trial site to complete the diary, citing reasons such as app incompatibility with their personal phone operating system, glitches after it was downloaded on their phones, and not wanting to use their own cellular data to operate the app on their phones. The electronic diary was reportedly not burdensome to complete on a daily basis (Table 3). Participants described an overall positive experience with the electronic app and the device they used, requiring only 2–4 min to complete. All participants stated the app was easy to learn and use.

## Discussion

FDA guidance on measuring and analyzing COVID-19–related symptoms in clinical trials includes recommendation of an evaluation to ensure the patient-reported outcome instrument’s basic comprehensibility and usability [5]. To follow this guidance, cognitive debriefing exit interviews with a subset (*n* = 25) of EPIC-SR clinical trial participants were conducted. Results indicate all 14 symptom diary questions were clear, were generally easy to answer using the response scales provided, and captured the participants’ experiences. Similarly, the 3 global items were generally easy to answer and perceived as clear by most participants and were interpreted as intended. The electronic app was easy to use, and the daily diary could be completed quickly and easily.

The 14 symptoms from the diary were the most frequently endorsed among those reported by all interviewees, supporting validity of the diary content. Additionally, the most frequently reported symptoms are generally similar to those reported by previous studies, although overall rates and relative frequencies for the reported symptoms differed [6–9], possibly due to differences in study populations and circulating strains at the time the studies were conducted [6–10].

Although some participants provided suggestions (e.g., expanding response options, adding comment fields), all confirmed it was easy to provide responses for each diary item. The few additional symptoms suggested by participants lends support to diary utility. A few participants noted having difficulties with global impression items (e.g., vagueness), particularly when trying to assess their overall symptom experience for multiple symptoms at their worst and over the past 24 h. This may result from the heterogeneous and inconsistent nature of COVID-19 symptoms.

Limitations of this analysis included that participants were selected from a clinical trial assessing symptomatic infection in those either fully vaccinated with ≥ 1 risk factor for severe disease or unvaccinated (or not vaccinated within the past 12 months) with no risk factors [3]. The convenience sampling approach, which recruited 25 participants from sites with high expected volumes, may also have introduced bias toward high-volume sites. Furthermore, generalizability of findings may be limited due to age-based differences in symptom experience (older adults were precluded), potential symptom reporting differences by sex (64% of participants were female), and cultural variation (interviews were conducted only in English-conversant individuals). These limitations potentially necessitate confirmation of applicability to real-world settings that include diverse age ranges and cultural backgrounds. However, standard practice for conducting cognitive debriefings validating new PRO measures is to use the same language as was used to develop the tool; separate linguistic validation processes are required for translations, exceeding analysis scope. Additionally, the current diary has not been assessed in individuals with post-acute sequelae of SARS-CoV-2 infection (i.e., long COVID), which presents with symptoms differing from acute infection [11, 12].

## Conclusions

Based on FDA guidance, results from this recommended analysis of the COVID-19 symptom diary and global impression items from the EPIC-SR study, with 14 diary symptoms capturing the most commonly experienced COVID-19 symptoms, validate the instrument utility for inclusion in clinical development and potential real-world clinical practice.

## Electronic supplementary material

Below is the link to the electronic supplementary material.

Supplementary Material 1

## Data Availability

Upon request, and subject to review, Pfizer will provide the data that support the findings of this study. Subject to certain criteria, conditions and exceptions, Pfizer may also provide access to the related individual de-identified participant data. See https://www.pfizer.com/science/clinical-trials/trial-data-and-results for more information.

## Abbreviations

CIOMS: Council for International Organizations of Medical Sciences
COVID-19: Coronavirus disease 2019
EPIC: Evaluation of Protease Inhibition for COVID-19
EPIC-SR: EPIC trial in patients at standard risk for severe disease
FDA: US Food and Drug Administration
GCP: Good Clinical Practice
ICH: International Council for Harmonisation
M^pro^: SARS-CoV-2 main protease
SARS-CoV-2: Severe acute respiratory syndrome–associated coronavirus 2
