# Supplementary material for: Assessing participants’ experiences with the COVID-19 symptom diary in a clinical trial
Source: J Patient Rep Outcomes. 2025 Aug 11;9:99. doi: 10.1186/s41687-025-00901-5 (PMC12339813; doi:10.1186/s41687-025-00901-5)
Supplement: Supplementary file 1 — Supplementary Material 1 [file 41687_2025_901_MOESM1_ESM.docx]

# Supplemental Appendix

## Clinical Site Participation and Interview Conduct

Of the 25 clinical sites that were selected to receive an invitation to participate in the interview substudy, 18 responded and completed a web-conference training session, and 10 sites provided recruitment logs, including patient contact information. Information for 53 potential interview participants was included on the recruitment logs; 25 ultimately completed exit interviews. Of the 28 patients who did not complete the exit interviews, 22 were not English-conversant (only Spanish) and 6 individuals discontinued the trial and/or were lost to follow-up. Overall, 22 (88.0%) of the 25 interviews were completed within 1 week after the trial participants’ Day 34 visits. Interviews for the remaining 3 (12.0%) participants occurred 9 to 20 days after their Day 34 visits because they had to reschedule or were difficult to contact initially.

## Interview procedures

Participants were contacted by telephone and/or email to schedule the interviews. All interviews were conducted via telephone following the methods and procedures outlined in the interview guide. Each interview was audio recorded and lasted from 45 to 60 minutes. At the beginning of each interview, the purpose was explained to each participant, and they were reminded that the interview was to be audio recorded and transcribed for use in preparing a written summary report of the interviews. In addition to the written consent obtained by clinical sites, verbal consent was obtained before the start of the recording and/or interview and again after the recording started, the participant was informed that recording has begun and asked to confirm their permission to continue.

Interviews began with a brief overview of the process and a few open-ended questions asking participants to describe their experiences with COVID-19, including how they were diagnosed and the symptoms they experienced. These general questions were followed by more targeted questions designed to gather information pertinent to the specific symptoms included in the diary. Next, specific to the cognitive debriefing process for the COVID-19 symptom diary and global impression items, participants were asked about their understanding of and ease of response to the questions and their perceived relevance of each of the diary and global impression items. All audio recordings of participant interviews were transcribed.

## Adverse event reporting

Potential safety events or product complaints were not actively solicited, ascertained, or evaluated as part of the interviews. However, if during an interview the interviewer became aware of any event (unanticipated symptom, condition, or issue) or product complaint experienced during the participant’s participation in the clinical trial, the interviewer forwarded the information to the participant’s clinical site within 24 hours.

## Interview Guide

Qualitative Interviews of Patients with Coronavirus Disease (COVID-19)

**Introduction**

**Interviewer introduces herself. Reminds participant of the purpose and format of the interview as described below and confirms consent to continue.**

[Note that this is a semistructured interview guide, not a script. It will guide the topics of discussion; other questions may also be asked and not all questions will be asked of all participants. The goal is to maintain a conversational approach.]

- We are interested in learning more about the symptoms you experienced with COVID-19, and specifically, the daily diary questions you completed throughout the trial. The information you share with us today will help the study sponsors to better understand the symptoms that individuals experience with COVID-19, as well as how to measure them in future trials.
- Your participation in the interview is completely **voluntary**, and you may end the discussion at any time. Of course, you should also feel free to ask us any questions you have or request a break in the interview at any time. We expect today’s interview to last about 45 minutes.
- With your permission, we will **audio record** today’s interview to make sure we do not miss any important information. The recordings will also allow us to create typed transcripts of the interviews. All information you provide to us will be kept confidential. While the transcripts will be provided to the study sponsor, all names and any other identifying information will first be removed from the documents.
- As we go along, please feel free to **speak openly** and share your thoughts freely. There are no wrong answers. You are the expert. We appreciate the opportunity to learn from your experiences and are truly grateful for your time.
- In today’s call, if you report a **side effect** that you experienced during the clinical trial, I will let the clinical site know. I may ask you some follow-up questions to fully understand what happened so I can share the details with the site for them to follow-up further with you, if needed.

**Before we begin, do you have any questions?**

**Okay, I’m going to go ahead and turn on my recorder.**

**START RECORDING*****REMEMBER TO TURN ON THE AUDIO RECORDER ONCE CONSENT IS PROVIDED. INTERVIEWER WILL VERBALLY NOTE STUDY ID AND INTERVIEW DATE***

*We have started the audio recording. Do we have your permission to continue with the interview?*

| 🞏 Yes | 🡪 | **CONTINUE** |
| --- | --- | --- |
| 🞏 No | 🡪 | **STOP INTERVIEW** |

**COVID-19 Signs and Symptoms**

**First, let’s talk about your initial experiences with COVID-19—more than one month ago.**

1. What types of signs or symptoms did you experience—even if you weren’t sure they were really related to COVID-19 at the time? [spontaneous report—before or after diagnosis; mark table below]
2. What symptoms or experiences, if any, prompted you to get tested for COVID-19?
3. After you started the trial, what did you experience? [spontaneous report of signs and symptoms; mark table below]

**I’m going to mention a few other symptoms and you tell me whether or not it is something you have experienced since you were diagnosed with COVID-19 and started the clinical trial.**

1. Did you experience [any/problems/differences] [if not mentioned above]

| [RECORD OF ALL SIGNS AND SYMPTOMS] | **Q1 - Q3 (🗸)** | **Q4 (🗸) [probed]** |
| --- | --- | --- |
| 1. Stuffy or runny nose |  |  |
| 2. Sore throat |  |  |
| 3. Shortness of breath (difficulty breathing) |  |  |
| 4. Cough |  |  |
| 5. Low energy or tiredness |  |  |
| 6. Muscle or body aches |  |  |
| 7. Headache |  |  |
| 8. Chills or shivering |  |  |
| 9. Feeling hot or feverish |  |  |
| 10. Nausea (feeling like need to throw up) |  |  |
| 11. Vomiting |  |  |
| 12. Diarrhea |  |  |
| 13. Your sense of smell |  |  |
| 14. Your sense of taste |  |  |
| [record other signs/symptoms below] |  |  |
|  |  |  |
|  |  |  |
|  |  |  |
|  |  |  |

**Cognitive Debriefing – COVID-19 Signs and Symptoms Diary**

**Next, we would like to review the COVID-19 signs and symptoms diary that you** **completed electronically for 28 days as part of the clinical trial.**

**First, the diary asked you about 10 symptoms. It asked you to rate the severity of each, at its worst, and over the last 24 hours. *(i.e.,*** *“What was the severity of your [symptom] at its worst over the last 24 hours?”)*

1. I’m going to read you each of these symptoms and I’d like for you to tell me what each means to you—or how you would define it using your own words.
   1. The first one was **Stuffy or runny nose**, what does that mean to you?
   2. Sore throat
   3. Shortness of breath (difficulty breathing)
   4. Cough
   5. Low energy or tiredness
   6. Muscle or body aches
   7. Headache
   8. Chills or shivering
   9. Feeling hot or feverish
   10. Nausea (feeling like you wanted to throw up)

**For each of these 10 symptoms…**

1. How easy or hard was it to think back over the past 24 hours?
2. Overall, how easy or hard was it to think about the symptom at its worst?
3. How easy or hard was it to rate the severity of the symptoms on the scale provided:

*None (I did not have this symptom), mild, moderate*, and *severe*?

1. Then, the diary asked you, *“How many times did you vomit (throw up) in the past 24 hours?”*
   1. How easy or hard was it to answer that question using the scale provided, from

*I did not vomit at all, 1-2 times, 3-4 times, and 5 or more time*s?

- 1. How easy or hard was it to think back over the past 24 hours to answer this question?
  2. Is there anything that could make this question clearer?

1. The next item asked you “*How many times did you have diarrhea (loose or watery stools) in the last 24 hours*?”
   1. How easy or hard was it to answer that question using the scale provided, from

*I did* not *have diarrhea at all, 1-2 times, 3-4 times, and 5 or more time*s?

- 1. How easy or hard was it to think back over the past 24 hours to answer this question?
  2. Is there anything that could make this question clearer?

**The final two questions asked about your sense of smell and taste in the last 24 hours.**

1. Sense of smell. What does that mean to you?
   1. How easy or hard was it to answer that question using the scale provided. The response options were as follows: *My sense of smell is THE SAME AS USUAL, my sense of smell is LESS THAN USUAL, and I have NO sense of smell*?
   2. How easy or hard was it to think back over the past 24 hours to answer this question?
   3. Is there anything that could make this question clearer?
2. Sense of taste. What does that mean to you?
   1. How easy or hard was it to answer that question using the scale provided. The response options were as follows: *My sense of taste is THE SAME AS USUAL, my sense of taste is LESS THAN USUAL, and I have NO sense of taste*?
   2. How easy or hard was it to think back over the past 24 hours to answer this question?
   3. Is there anything that could make this question clearer?

**We have now talked about all of the symptoms in the COVID-19 Diary.**

1. Overall, how well does this diary describe and measure the signs and symptoms you experienced with COVID-19?
2. Is there anything important about your experience, with any of the 14 signs and symptoms in this diary, that is not captured?
3. Among all of the signs and symptoms in this diary that you experienced, what was the most bothersome for you and why? [remind patient of the 14 symptoms if needed]
4. Based on your experience, are there any COVID-19 signs and symptoms, that are missing from this diary?

**Cognitive Debriefing – Global Impression Items**

**Next, we would like to review 3 additional items that were also included in the electronic diary you completed as part of the trial. I will read each question and then ask you about it.**

1. The first one was “***In the past 24 hours, have you returned to your usual health (before your COVID-19 illness)*?”** and you were asked to answer yes or no.
   1. In your own words, what is this question asking you?
   2. What does “your usual health” mean to you?
   3. How easy or hard was it to think about the last 24 hours and answer this question?
2. The second one was “***In the past 24 hours, have you returned to your usual activities (before your COVID-19 illness)*?”** and you were asked to answer yes or no.
   1. In your own words, what is this question asking you?
   2. What does “your usual activities” mean to you?
   3. How easy or hard was it to think about the last 24 hours and answer this question?
3. The last one was “***In the past 24 hours, what was the severity of your overall COVID-19-related symptoms at their worst?****”* and you were asked to answer as none, mild, moderate, or severe.
   1. In your own words, what is this question asking you?
   2. How would you describe each of the responses: *none, mild, moderate, and severe* overall symptoms of COVID-19?
      1. What is the difference, if any, between none and mild? Mild and moderate? Moderate and severe?
   3. How easy or hard was it to think about the last 24 hours and your overall symptoms at their worst to answer this question?

**Feedback on Electronic Diary Experience (10 minutes)**

**As we reach the end of this interview, I would like to get your thoughts on completing all of these questions on the electronic or handheld device.**

- Overall, what did you think of the electronic device?
- Was it easy or hard for you to learn how to complete the diary on the electronic device? How so?
- Once you learned the electronic device, was it easy or hard for you to use it to complete the diary? Why?
- Do you have any suggestions on how we could make the electronic device easier to use?

**Conclusion**

Thank participant again and end interview!

## Supplementary Table S1. Most Bothersome COVID-19 Diary Symptoms

| **Most bothersome symptom** | **Participants reporting symptom, n (%)^a^**  **(n=20)** | **Quotes describing why the symptom was the most bothersome** |
| --- | --- | --- |
| Low energy or tiredness | 5 (25.0) | “[N]ot being able to shower properly”  “[H]ard to push past when it was really bad to do schoolwork”  “[D]idn’t have energy to do anything…just lay on the couch”  “[W]asn’t able to do anything/take care of self" |
| Lost sense of taste | 4 (20.0) | “Not being able to taste food made it hard for me to eat and I get a lot of excitement from eating”  “[A]ffected appetite” |
| Lost sense of smell | 3 (15.0)^b^ | “I love to cook and not being able to smell the food you’re cooking…that’s a bummer”  “[L]ost appetite”  “[D]idn’t know if I’d recover it at all…took almost 2 weeks to come back” |
| Headache | 3 (15.0) | “[I]t was debilitating, had to sit in the dark”; “stayed in bed and wasn’t able to get up and do any regular activity”  “[P]revented working” |
| Cough | 2 (10.0) | “[C]ough was severe and lingered”  “[D]idn’t want to cough in public/self-conscious” |
| Stuffy or runny nose | 1 (5.0)^c^ | “[T]riggered cough” |
| Shortness of breath  (difficulty breathing) | 1 (5.0) | “[P]revented exercising, doing chores, regular routine of the day” |
| Muscle or body aches | 1 (5.0) | “[I]t was hard to push through” |
| Chills or shivering | 1 (5.0) | “[B]ecause nothing could be done [to alleviate]” |
| Feeling hot or feverish | 1 (5.0) | “[D]idn’t feel like doing anything” |
| Vomiting | 1 (5.0) | “[I]t’s no fun throwing up” |
| Nausea | 0 | – |
| Sore throat | 0 | – |
| Diarrhea | 0 | – |

^a^Although the sample was n=20, the number of reports was n=23 because 3 patients reported 2 most bothersome symptoms.

^b^ Two participants reported “loss of sense of smell” to be equally bothersome as lost sense of taste.

^c^ One participant reported “stuffy or runny nose” to be equally bothersome as cough.

## Supplementary Table S2. Description of COVID-19 Symptom Experiences^a^

| **Symptom** | **Definition/description in participants’ own words** |
| --- | --- |
| Stuffy or runny nose | Congestion, nose dripping like a faucet, can’t breathe through nose, snot running down face, constantly blowing nose, nose leaking, lots of boogers, clogged nose |
| Sore throat | Dry, achy, burning sensation, scratchy, painful, tender, tickly, tightness, trouble swallowing, hard time eating food |
| Shortness of breath (difficulty breathing) | Hard time getting enough air in or out, not being able to breathe normally, struggling to catch breath, chest tightness, heaviness in lungs, wheezing, gasping for air |
| Cough | Action to clear tickle/itch in the back of throat, body trying to push out mucus from throat, violently expelling air |
| Low energy or tiredness | Weakness, more tired than normal, fatigue, exhaustion, extreme amount of effort to do anything, always wanting to sleep, no strength to do anything, no energy to get through daily activities |
| Muscle or body aches | Soreness, stiffness, whole body hurting, dull ache, tenderness, throbbing muscles, pain associated with movement, body hurts like after going to the gym |
| Headache | Sharp pain in head, pressure feeling in head, like a migraine, felt like head was about to blow up, soreness in the head, tension in head, throbbing in head |
| Chills or shivering | Unusually cold in relation to the temperature, chilly even when wrapped up in a blanket, twitching of body, goosebumps, shaking, chattering of teeth, being abnormally cold, cold to the bone |
| Feeling hot or feverish | Feeling overheated, hot flash, dry tongue, body warm to touch, head feeling hot, sweating |
| Nausea (feeling like need to throw up) | Queasiness, dry heaving, upset stomach, feel of wanting to hurl, irritated stomach, feeling sick to the stomach |
| Vomiting | Throw up, food forcibly coming out of mouth, stomach rejecting everything eaten, contents of stomach come out of mouth |
| Diarrhea | Watery feces, loose poop, watery stool, frequent and watery bowel movements, cramping |
| Sense of smell | Ability to pick up on scents in the environment, ability to smell, using the nose to identify things, aromas gotten through the nose, ability to differentiate between different smells, perceiving odors |
| Sense of taste | Ability to taste, ability to recognize the flavor of something in your mouth, working taste buds, ability to differentiate between flavors |

^a^Responses are based on the phrases used by participants when asked to describe the symptoms in their own words.
